# Dysbiotic microbiota interactions in Crohn’s disease

**DOI:** 10.1080/19490976.2021.1949096

**Published:** 2021-07-27

**Authors:** Esther Caparrós, Reiner Wiest, Michael Scharl, Gerhard Rogler, Ana Gutiérrez Casbas, Bahtiyar Yilmaz, Marcin Wawrzyniak, Rubén Francés

**Affiliations:** a Dpto Medicina Clínica, Universidad Miguel Hernández, San Juan De Alicante, Spain; b Iis Isabial, Hospital General Universitario De Alicante, Alicante, Spain; c Department for Biomedical Research, Department of Visceral Surgery and Medicine, Bern University Hospital, University of Bern, Bern, Switzerland; d Department of Gastroenterology and Hepatology, University Hospital of Zurich, University of Zurich, Zurich, Switzerland; e CIBERehd, Instituto De Salud Carlos III, Madrid, Spain

**Keywords:** Crohn’s disease, microbiota, inflammation, fibrosis, dysbiosis, bacterial translocation

## Abstract

Crohn’s disease (CD) is a major form of inflammatory bowel disease characterized by transmural inflammation along the alimentary tract. Changes in the microbial composition and reduction in species diversity are recognized as pivotal hallmarks in disease dynamics, challenging the gut barrier function and shaping a pathological immune response in genetically influenced subjects. The purpose of this review is to delve into the modification of the gut microbiota cluster network during CD progression and to discuss how this shift compromises the gut barrier integrity, granting the translocation of microbes and their products. We then complete the scope of the review by retracing gut microbiota dysbiosis interactions with the main pathophysiologic factors of CD, starting from the host’s genetic background to the immune inflammatory and fibrotic processes, providing a standpoint on the lifestyle/exogenous factors and the potential benefits of targeting a specific gut microbiota.

## 1. Dysbiosis and bacterial translocation in CD

The intraindividual intestinal microbiota profile is extremely diverse and relatively stable over years with some minor reversible disruptions. Bacteria from Bacteroidetes (~15-50%) and Firmicutes (~20-50%) phyla with fair deviation and to a lesser extent and from Actinobacteria (<5%) and Proteobacteria (<10%) phyla constitute the main taxa in the human gut; however, there is a great diversity at lower taxonomic levels^1^. Healthy conditions lacking proinflammatory responses against commensal bacteria underlines the mutualism and clearly defined lines of communication between the microbiome and the host. Many features of the modern lifestyle have a direct fingerprint on the gut microbial profile; those include medications, diets high in refined carbohydrates, illness, hospitalization, surgery, smoking, alcohol abuse, processed foods low in fermentable fibers, and chronic stress.^2^

Host–microbial interactions are detrimental in CD patients with dramatic changes at strain levels and reduced species richness as well as many defined microbial metabolites^3–5^ compared to non-IBD healthy subjects (Table 1). Some of these alterations are already present at the earliest stage in pediatric, treatment-naïve patients^4,19^ as well as in healthy first-degree relatives^10^ of CD patients. Of note, the gut microbiota of CD patients with active disease is comparatively more unstable over time as compared to CD patients with inactive disease or non-IBD healthy individuals.^21^ In these patients, the blooming of Proteobacteria, appearance of *Fusobacterium*, and reductions in Clostridia cluster IV of anaerobic bacteria are reported.^1,22^

**Table 1.** **Microbial alterations in CD patients**. Relative changes in abundance of taxa in CD patients for Actinobacteria (A), Bacteroidetes (B), Firmicutes (F), *Proteobacteria* (P), Fusobacteria (Fu), and *Euryarchaeota* (E) phyla are categorized according to increases or reduction when compared to non-IBD subjects

Studies Reporting Reduced Taxa in CD	Studies Reporting Increased Taxa in CD
Firmicutes (F)^6–8^	Bacteroidetes (B)^7–9^
F. prausnitzii (F) ^1,3,4,6,9–15^	Bacteroidaceae (B)^7^
Lachnospira (F)^1,6,9,15^	R. gnavus (F) ^3,7,9,11,14–18^
Roseburia (F) ^1,3,4,12,14,15^	Oscillospira (F)^1,9^
Lachnospiraceae (F)^15^	[Eubacterium] (F)^9^
Blautia (F)^1,15,19^	Enterobacteriaceae^1^
Bifidobacterium^4,10,12,15,19^	Bifidobacterium (A)^9^
Clostridium (F)^1,3,8^	Clostridium (F)^9^
Clostridia (F)^8,19^	Enterobacter (P)^7^
C. coccoides (F)^13^ and C. leptum (F)^13^	Enterococcaceae (F)^4^
Prevotella^3^	^+^Veillonellaceae (F)^19,20^
Erysipelotrichales (F)^19^	Proteobacteria (P)^1,8^
Dorea (F)^1,19^	Enterobacteriaceae (P) ^3,7,12,15,18–20^
Anaerostipes^3^	E. coli (P) ^3,14,19^
Ruminococcaceae (F)^3,4,19^	Pasteurellaceae (P)^19^
Oscillospira (F)^3,9,19^	Atopbobium (A)^20^
Christensenellaceae (F)^3^	^+^Fusobacteriales (Fu)^3,12,19,20^
D. invisus* (F)^10,11,19^	Escherichia (P)^6^
Bacteroides (B)^1,7,9,19^	
Alistipes (B)^14^	
Actinomycetaceae (A)^17^	
C. aeroffaciens (A)^10^	
Methanobrevibacter (E)^3^	

Critically, many studies relate to stool samples but mucosal and mucus-associated microbiota has been recently shown to harbor consortia that are different from the fecal microbiota in terms of abundance, metabolic functioning, behavior, and replication.^23^ Thus, this makes the mucus layer of particular interest in CD which has not been exhaustively characterized. In fact, several key functional pathways have been delineated being activated differently in IBD patients,^24^ but deep metagenomic and metatranscriptomic investigations focusing specifically on CD are relatively scarce.^11^ Predominant transcription of pathways by individual microbes within a host are known, and thus, loss of these organisms in CD can have more fare-reaching consequences than indicated by their genomic abundance.^11^ Even though many microbial organisms exhibited concordant DNA and RNA abundances, it is also reported that species-specific biases in the transcriptional activity reveals predominant transcription of pathways by individual microorganisms per host.^11^ For instance, *Dialister invisus* is metagenomically present with relatively reduced abundance; however, it does transcriptionally loss of the gene function.^11^ For example, all ^+^-marked species in Table 1 are main producers of H_2_S through fermentation of sulfur-containing amino acids. Indeed, these genera have been suggested to predict the severity of CD (in conjunction with the concomitantly observed decrease in mitochondrial H_2_S detoxification capacity).^20^ Overall, it is important to include metagenomic and metatranscriptomic readouts that allow us to analyze both the activity and the presence of gut microorganisms, which ultimately provide better insight into the role of the microbiome in IBD.

Besides how each community member is abundant and functions in an intestinal ecological niche, it is important to understand their ecological roles which are fundamentally vital for the whole health biodiversity. The co-occurrence associations are beneficial to infer effects between taxa within the intestinal tract and are a key point to mechanistically examine the community structure and maintenance. Co-occurrence analysis by *Yilmaz et al*. revealed what could be considered as keystone species within the ecosystem of CD patients representing the most influential taxa such as *Faecalibacterium* and *Ruminococcus*.^1^ Together with *Lachnospira, Blautia, Dorea, Coprococcus, Roseburia, Oscillospira*, and *Bilophila, Faecalibacterium* and *Ruminococcus* build up the most prominent and influential taxa cluster in CD disease groups. Alterations of taxa within this network also characterize the risk of later disease recurrence of patients in remission after the active inflamed segment of CD has been surgically removed. The robustness of the microbial network as an interrelated cluster has been observed to associate with stable remission, whereas a loose structure of the microbial community and increases in abundance of *Enterobacteriaceae* is present in relapsing CD.^25^ In sum, the microbiota being present after surgery and/or lack of reestablishing a healthy robust cluster seems to be emblematic for the susceptibility of the patient to acquire CD manifestation again.

One important aspect that researchers still could not completely resolve is whether the disruption of the network leading to the dysbiosis is a cause or consequence of inflammation in CD. Inflammation is able to disrupt the stability of the microbial composition,^26^ and as a matter of fact, it has been proposed to drive dysbiosis and bacterial invasion in murine models of ileal CD.^27^ In addition, the extent of dysbiosis diminishes with decreasing inflammation and mucosal healing due to the beneficial effects of TNF inhibitors or exclusive enteral nutrition on patients underlining the potential effect of inflammation per se on the gut microbiota.^28^ However, it appears that the microbial compartment primarily and causatively contributes at least partly to inflammation when additional host factors are present since in animal experiments, i) dysbiotic gut microbiota can transmit Crohn’s like ileitis in mice independent of failure in antimicrobial defense^29^ ii) IL-2 and IL-10 deficient mice which are prone to develop colitis are protected when raised in germ-free conditions^30,31^ iii) susceptibility to CD is transmissible by stool microbiota of CD patients was shown in humanized gnotobiotic mice,^31^ and iv) gut contents elicit post-operative recurrence of CD in the terminal ileum proximal to the ileocolonic anastomosis after ileal resection.^32^ Thus, it is reasonable to propose that dysbiosis is also linked to treatment failure and postoperative recurrence in CD. Even though it is still not certain whether or to which extend this link is fundamental, one can argue that the failure to treatment lies within the underlying microbiota remaining as permanent sequelae in refractory CD which thus, can represent a promising target for treatment and/or preventive measures.^33^

**Figure 1.**
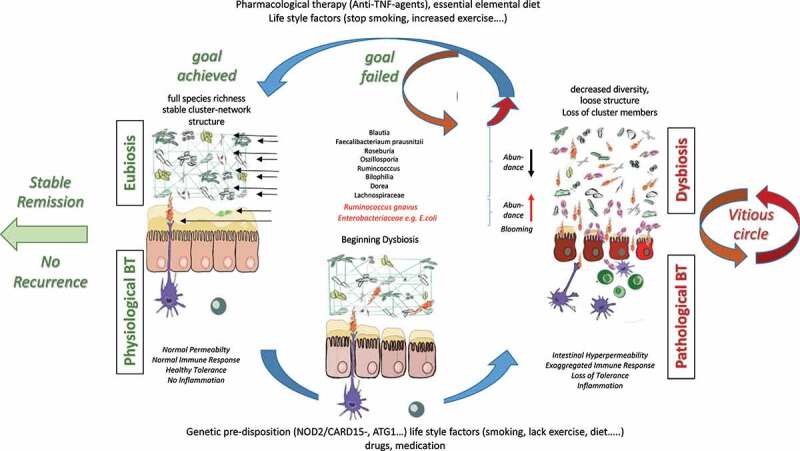
**Interaction of microbiome, intestinal barriers, and translocating bacteria in healthy conditions and active CD**. Eubiosis is characterized by stable cluster networks and full species richness which in concert with fully active healthy epithelial and secretory barriers (namely normal intestinal permeability) and normal immune responses to physiological bacterial translocation is the basis for stable remission. The proposed hypothetical scenario on pathophysiology of Crohn’s disease being at least partly primarily due to dysbiosis and loss of cluster network induced in individuals with genetic pre-disposition and/or by lifestyle/exogenous factors is visualized at the bottom. With increasing intestinal hyperpermeability and exaggerated pro-inflammatory immune response inflammation perpetuates fueling into a vitious circle of pathological bacterial translocation and further aggravation of dysbiosis. Thus, the goal of treatment put forward is restoration of eubiosis being the basis for stable remission or if not achieved for recurrence

Long-term effects of the bacterial network disruption with increased abundance of pathobionts within the gut might also influence the intestinal hyperpermeability even in the quiescent states of disease without any on-going inflammation.^34^ More recently and revealing, increases in intestinal permeability have been evidenced to determine later development of CD and thus, contributing to pathogenesis.^35^ The path of viable bacteria and/or their products such as outer membrane vesicles, lipopolysaccharides, peptidoglycans, muramyl-dipeptides, and bacterial DNA from the lumen through the gastrointestinal mucosa to normally sterile tissues, such as the mesenteric lymph nodes (MLN) and extra-nodal sites is referred to as bacterial translocation (BT).^36,37^ The link between dysbiosis and inflammation can be considered to be any pathological increase in BT due to alterations in intestinal hyperpermeability (Figure 1). Numerous studies have sampled MLN of patients suffering from IBD and conditions requiring surgery using culture-dependent techniques. Pathological bacteria translocation (PBT) to MLN has been realized for long in CD.^36,38^ *Ambrose et al*. showed that cultured bacteria from nodes were higher in numbers in involved CD segments than uninvolved ones where these bacteria were almost absent in control subjects.^36^ *Takesue* and colleagues obtained similar trends from isolated bacteria from MLN of subjects (CD patients (48%) and control (15%))^39–41^

Pathological increases in BT in contrast to physiological BT reflects sustained increases in quantity (rate and/or degree) and/or type (composition) of translocating agents. In that regard, as the first step across the epithelial layer increased adherence and endocytosis into enterocytes of commensal bacteria, particularly *E. coli* is observed in CD patients.^39,42^ Although any specific microorganism was absent in bacterial DNA positive nodes of CD patients or associated mucosa, *Escherichia/Shigella* (CD – 75% and healthy subjects – 60%) was detected at high levels in patients with DNA-positive lymph nodes. Of note, patients with terminal ileal CD had a greater proportion of *E. coli*.^39^ In fact, not only for *E. coli* but per se an increase in presence of adhesins and other virulence factors has been demonstrated in feces from CD patients.^12^

The follicle-associated epithelium lining Peyer’s patches is characterized as the site of increased translocation of bacteria to the underlying lymphoid tissue. Earliest observable lesions of recurrent CD are frequently microscopic erosions at the FAE, and an increased load of commensal bacteria at this vulnerable and inductive site of mucosal immunity has been reported in CD.^42^ Moreover, bona fide invasive pathogens, such as *Salmonella* and *Shigella*, are unable to invade via normal colon epithelial cells. However, they enter via the specialized M cells which lack fuzzy glycocalyx within the dome epithelium overlying Peyer’s patches in the distal ileum and lymphoid follicles in the colon.^43^ In addition, one of the most translocating bacterial strains is adherent-invasive *E. coli* (AIEC) pathovar which is increased in mucosa of CD patients.^44^ AIEC of CD patients lacking conventional pathogenicity genes might penetrate through M cells which fits well with evidence suggesting that the earliest lesions in CD occur at PP in the ileum and lymphoid follicles in the colon.^45^ Moreover, resident dendritic cells accumulate in an increased number in the subepithelial dome of Peyer’s patches in ileal CD and present with pronounced propensity to internalize translocated *E. coli*.^46^ These *E. coli* also cause IL-8 to be released from intestinal epithelial cells and induces granuloma formation after internalization by cultured macrophages.^47^

Another important target site for PBT from the gut is the mesenteric adipose tissue when the leaky gut occurs in CD.^48,49^ BT to mesenteric adipocytes i) occurs at a rate similar to the translocation to MLN;^50^ ii) could represent a bacterial reservoir^51^ iii) stimulating visceral fat for CRP-production;^50^ and iv) in terms of the microbiota profile dependent on the clinical status in CD patients.^51^ In addition, *Ha et al*. characterized the subset of mucosal-associated gut bacteria that consistently translocated and remained viable in “creeping fat” defined as an expansion of mesenteric adipose tissue around the inflamed and fibrotic intestine in CD ileal surgical resections.^52^ Translocation of *C. innocuum* was identified as a signature of this consortium i) with strain variation between mucosal and adipose isolates and ii) stimulating tissue remodeling via M2 macrophages leading to an adipose tissue barrier that serves to prevent systemic spread of translocated bacteria.^52^ Thus, in conjunction with the well-known cytokine production and immune cell infiltration in adjacent tissue in intestinal inflammation, the mesenteric fat might cooperate with the gut in a proinflammatory feedback loop, generating a specific microbiome signature in this tissue to influence the CD status and/or disease progression.^51,52^

PBT to the systemic compartment reflected by detection of bacterial DNA in serum has recently gained much attention^53,54^ revealing Enterobacteriaceae as a major source of bacterial DNA^53^ within actively smoking CD patients harboring NOD2-, CARD15- and ATG16L1-variants.^55^ Hence, these genetic risk factors for CD not only inhibit beneficial effects of microbial products during physiological BT to keep up mucosal tolerance but also drive pathology. PBT to the blood stream is evident almost twice as frequent in patients with clinically active disease as compared to patients with recurring disease.^55^ In fact, bacterial DNA translocation was significantly and independently related to disease activity. In a prospective, multicenter study on CD patients with CDAI<150, bacterial DNA translocation to serum was demonstrated to represent an independent risk factor for relapse and hospitalization within 6 months.^53^ Potential mechanisms explaining the impact of BT on the disease activity and aggravating course of disease are the reported consequences of translocating bacterial DNA on systemic and local inflammatory response. Bacterial DNA in serum has been found to associate with increased antimicrobial peptide (β-defensin 2, cathelecidin LL-37) and pro-inflammatory cytokine levels in CD patients in a concentration-dependent manner.^54^ In addition, the increased proinflammatory activity in the presence of bacterial DNA translocation is particularly pronounced in CD patients carrying mutated variants of NOD2/CARD15. Indeed, neutrophils from CD patients with variant NOD2-status release significantly more TNF when exposed to *E. coli* DNA *in vitro* as compared to CD patients with wild-type NOD2 status.^55^ Moreover, the presence of bacterial DNA in serum seems to decrease the availability of free anti-TNF in CD patients treated with biologicals.^55^ It is tempting to speculate that this is caused by pronounced TNF secretion in response to bacterial DNA in CD with NOD2-variant genotype due to faster consumption of free anti-TNF.^55^ Thus, PBT in subgroups of CD patients could (at least partly) cause the requirement of intensified anti-TNF therapy.

The observation that PBT per se disrupts the epithelial barrier and increases intestinal permeability^56,57^ strongly indicates the perpetuating potency of bacterial translocation as driving force of a ***vicious cycle*** in CD (Figure 1). This even more though considering that AIEC type 1 pili-mediated interaction with cell-adhesion molecules abnormally expressed in the quiescent phase of CD may disrupt intestinal barrier integrity before the onset of inflammation.^56^ Accumulating evidence shows that pathobionts by virtue of their possession of adhesion mechanisms and secretion of proteases, may have a causative role in CD. In CD, they most likely invade the tissue, perhaps as a result of defective clearance by neutrophils and macrophages. In addition, inflammation itself and disruption of the epithelial barrier increases intraluminal oxygen availability which leads to an outgrowth of pathobionts. This example highlights the complex interaction between intestinal barrier, gut microbiota and BT which when delineated in more detail will ultimately lead to improvements in preventive and therapeutic measures in CD.

## 2. Interaction between microbiota, genetics, and epigenetics in CD

IBD risk genes alter the gut microbiota composition

As stated above, genetic risk factors influence the interaction between gut microbiota and mucosal microenvironment in CD. Genetics is a well-known susceptibility component for IBD development, with more than 240 genomic IBD susceptibility loci identified in the GWAS.^58^ Data analyzed from several GWAS identified 30 CD-specific loci, 23 UC-specific loci, and 110 loci that play a role in both forms of IBD.^59^ Interestingly, genes involved in the epithelial barrier function were found to be more associated with UC and genes engaged in cellular innate immunity with CD. Additionally, more than 8% of variance in UC susceptibility and more than 13% of the variance in CD susceptibility might be explained by currently identified genetic variants.^60^

The host genetics, similar to environmental factors, have been shown to influence the gut microbiome composition and diversity^61^ (Table 2). NOD2, the first IBD susceptibility gene identified, is a NOD-like receptor that binds bacterial muramyl dipeptide.^72^ Studies involving NOD2-deficient mice established that the absence of NOD2 increases the relative abundance of *Bacteroidetes* and impairs the intestinal microbiota in several mouse models.^73,74^ Similarly, NOD2-risk allele-related increases in *Enterobacteriaceae* family was observed in three IBD cohorts.^75^

**Table 2.** Influence of IBD-associated genes on the microbial composition

Gene symbol	Influence on microbial composition	Reference
***NOD2***	*Enterobacteriaceae, Bacteroidetes*	^62^
***ATG16L1***	*Fusobacteriaceae, Bacteroidaceae, Enterobacteriaceae*	^63^
***FUT2***	decreased microbial diversity and changes in several taxa	^64^
***SLC39A8***	*Anaerostipes, Coprococcus, Lachnospira*	^65^
***CARD9***	*Citrobacter rodentium* infection More intestinal fungi	^66^
***NLRP12***	*Lachnospiraceae, Erysipelotrichaceae*	^67^
***TNFSF15***	*Prevotella*	^68^
***BANK1, EFR3B, IL1R2, POMC***	*Β-diversity*	^69^
***IL6***	*Helicobacter pylori*	^70^
**11 functional genetic variants in genes**: *NOD2, CARD9, ATG16L1, IRGM* **and** *FUT2*	decrease in abundance of butyrate producing bacteria	^71^

NOD2 (Nucleotide-binding Oligomerization Domain Containing protein 2), ATG16L1 (Autophagy-Related 16 Like 1), FUT2 (Fucosyltransferase 2), SLC39A8 (Solute Carrier Family 39 Member 9), CARD9 (Caspase Recruitment Domain Family Member 9), NLRP12 (NLR Family Pyrin Domain Containing 12), BANK1 (B Cell Scaffold Protein with Ankyrin Repeats 1), EFR3B (EFR3 Homolog B), IL1R2 (Interleukin 1 Receptor Type 2), POMC (proopiomelanocortin), *IRGM* (Immunity Related GTPase M), IL6 (Interleukin-6).

Next, ATG16L1, a gene identified by GWAS studies as IBD risk gene, regulates the development of T cells and cell autophagy.^76^ In mouse models, deletion of ATG16L1 triggers spontaneous intestinal inflammation with severely reduced CD4 + T cells.^77^ In ATG16L1 homozygous CD patient’s, enrichment of *Fusobacteriaceae, Bacteroidaceae*, Enterobacteriaceae in ileal tissue was observed as compared to control patients.^63^ Fucosyltransferase 2 (FUT2), an enzyme that regulates intestinal epithelial cells-microbe interaction is another factor that lost was found to increase susceptibility to CD development. Patients lacking functional FUT2 alleles have abnormal mucosal barrier with decreased microbial diversity and changes in several taxa.^64,78^ Additionally, the association between risk locus SLC39A8 and the abundance of *Anaerostipes, Coprococcus*, and *Lachnospira* was found in CD patients.^65^

Furthermore, combined genetic risk scores from 11 functional genetic variants associated with IBD susceptibility were directly involved in the bacterial composition in the gut and were associated with a decrease in the abundance of butyrate-producing bacteria.^71^

Interaction between the microbiota and epigenetics in IBD

Genetic susceptibility to develop disease might explain only part of disease risk and its heritability.^79^ The contribution of genetic risk alone to disease development was suggested to be around 20%.^80^ Epigenetic regulation of gene expression was proposed to play an additional role in the development and control of IBD.^81^ According to the current definition, epigenetic mechanisms are all heritable alternations of gene expression that are caused independently of changes in the primary DNA sequence.^81^ DNA methylation, histones modifications modulating chromatin structure, microRNA interference, and positioning of nucleosomes are the main epigenetic mechanisms that control gene expression.^82^

DNA methylation is a process characterized by the covalent addition of a methyl group to 5` carbon of cytosines in cytosine-guanine dinucleotides (CpG).^83^ This process is catalyzed by DNA methyltransferases (DNMTs). DNMT1, DNMT2, DNMT3a, DNMT3b, and DNMT3L are five members of DNMT protein family.^84^ As a result, hypermethylation of CpG island in promoters is transcriptionally repressive and genes are silenced.^85^

In addition, genes expression might be regulated by posttranslational histone modifications that include acetylation, methylation, ubiquitination, phosphorylation, sumoylation, and citrullination of histone tails.^86^ Acetylation is the best characterized process, and histone acetyl transferases (HATs) are the enzymes responsible for addition of acetyl groups to lysine residues in histones. Histone acetylation is associated with chromatin opening and transcriptional gene activity. On the other hand, histone deacetylation by histone deacetylases (HDACs) triggers chromatin compacting and gene silencing.^87^

The epigenetic machinery is influenced by environmental factors including intestinal microbiota and their metabolites. The influence of bacteria on the epigenetic control of gene expression is well characterized for SCFAs, a bacterial metabolite formed during anaerobic fermentation of dietary fibers. SCFAs, namely acetate, propionate, butyrate, produced by Firmicutes and Bacteroides phyla, inhibits HDAC activity.^88^ Interestingly, reduced numbers of SCFA produces have been observed in IBD patients.^89^ There are two possible mechanisms of SCFA action. The first mechanism involves regulation of naïve CD4 + T cells differentiation into T regulatory cells (T regs). It has been shown that acetylation of H3 histone within Foxp3 loci that is required for T regs differentiation is increased by butyrate.^88^ The second mechanism involves macrophages, the most abundant lamina propria cells. It has been shown that ther production of IL-6 and IL-12 from lipopolysaccharide stimulated macrophages was downregulated by addition of butyrate.^90^ Downregulation of proinflammatory cytokines produced by macrophages suggests that butyrate might induce hyporesponsiveness of macrophages and maintain tolerance in the gut.

As the epigenetic signature is profoundly cell specific, another cell type – intestinal epithelial cells (IECs), gained significant attention in studies focused on epigenetic regulation in relation to IBD. In their work on IECs from CD patients and mouse modes, *Denizot et al*. suggested that demethylation of CEACAM6 gene promoter in IECs correlated to high protein expression and favored gut inflammation mediated by colonization by AIEC. For the first time, due to epigenetic machinery, diet and the presence of methyl-donor ingredients were linked to the microbiota and inflammation.^91^

The influence of the microbiota on transcriptome and DNA methylation in IECs during postnatal development was investigated by *Pan at el*. Interestingly, differences in DNA methylation that are depended on the microbiota were detected early after birth, in contrast to transcriptome, where bacterial influence increased over time.^92^ Moreover, authors reveal 126 genomic loci where the presence of the intestinal microbiota was associated with combined differential RNA transcription and DNA methylation. The most important conclusion from this study is that during development, microbiota modulates epithelial cells transcriptome and functionally methylate genes and, on long term, change their expression signature in IEC.^92^

In summary, epigenetic modifications connect genetic susceptibility with environmental factors via the intestinal microbiota. As epigenetic changes are reversible in nature, from clinical perspective, it is necessary to understand how it would be possible to control or even reverse disease associated changes through epigenetic mechanism.

## 3. Dysbiotic microbiota effect on local and systemic inflammation in CD

**Figure 2.**
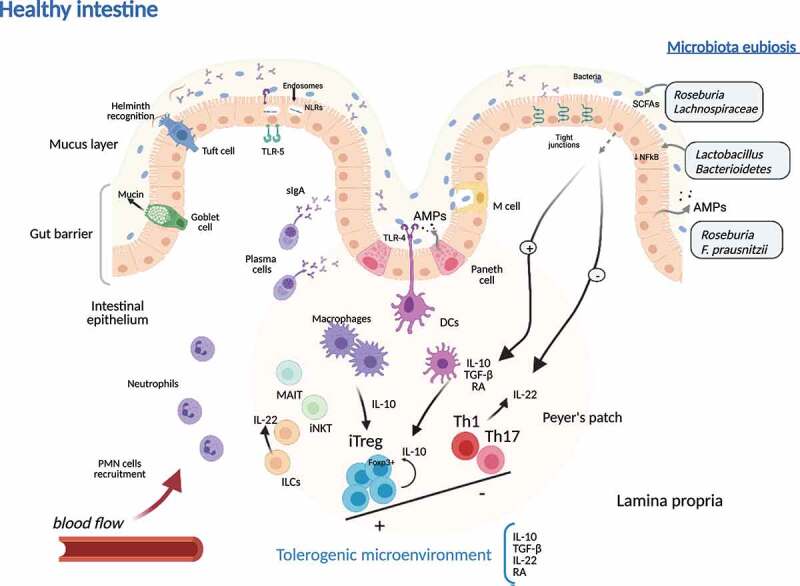
**Tolerogenic microenvironment during microbiota eubiosis in healthy intestinal tissue in Crohn’s disease**. In healthy colonic tissue, the gut barrier is made up of a thick mucus layer containing mucin produced by Goblet cells, sIgA produced by plasma cells, AMPs secreted by epithelial cell, and the cellular immune system (ILCs, MAIT cells, NKTs, macrophages, dendritic cells and T cells), mainly organized in Peyer’s patches. DCs and macrophages produce anti-inflammatory mediators that promote iTreg Foxp3+ expansion. Moreover, the eubiotic microbiota contributes to reduction of proinflammatory substrates induction (reduced activation of NFkB in epithelial cells) and produces SCFAs and subsequent inhibition of proinflammatory (Th1 and Th17) cell lineage activation. iTregs together with eubiotic microbiota are responsible of the predominant immunotolerance in intestinal tissue microenvironment, while ILC-derived IL-22 contributes to keep gut barrier integrity. sIgA, soluble IgA; AMPs, antimicrobial peptides; ILC, innate lymphoid cells; MAIT, mucosal associated invariant T cell; iNKT, invariant Natural Killer T cell; DC, dendritic cell; iTreg, induced regulatory T cells; NFkB, nuclear factor kappa-B; Th, T helper cell; SCFAs, short chain fatty acids, PMN, polymorphonuclear cells; TLRs, Toll-like receptors; NLRs, Nod-like receptors; IL, interleukin; RA, retinoic acid; TGF-β, transforming growth factor-β

As shown, the genetic background may influence the microbiota composition, which, in turn, tightly shapes the immune pathogenic mechanisms present in CD. During homeostasis, thus immune quiescence, microbial members of the commensal microbiota contribute to the maintenance of the gut barrier integrity and the optimal mucosal composition. They collaborate in the prevention of microorganisms crossing through the intestinal epithelium^93^ (Figure 2).

Regulatory T (Treg) cells interact with the microbiota, and their expansion is controlled by products of commensal bacteria,^94^ and their balance with adaptive Th17 cells (main productors of IL-17 and IL-22) and the gut microbiota is directly implicated in illness developing or worsening.^95^ As an example, *Roseburia* and taxa from *Lachnospiraceae* family are in charge of producing short-chain fatty acids (butyrate, acetate, and propionate) from fermentable dietary fiber as a carbon supply for intestinal colonocytes and differentiation of Tregs.^88,96^ Also, polysaccharide-A (PSA) of *Bacterioides fragilis* induces IL-10-expressing Foxp3+ Treg cells,^97^ and Toll-like receptor (TLR)-2 internalization of PSA by dendritic cells induces the subsequent differentiation of IL-10-producing Treg cells in the colon.^98,99^ Moreover, *Roseburia* can improve the innate response in the gut by inducing antimicrobial peptide production and increasing the gut barrier function.^100^ Also, *Bacteroidetes* and *Lactobacillus spp*. can block NFκB signaling pathway, reducing proinflammatory microenvironment in the intestine. In vitro experiments using intestinal cell lines, *Faecalibacterium prausnitzii* can inhibit NFκB pathway through the microbial anti-inflammatory molecule MAM.^101^ Moreover, dendritic cells production of IFN-γ and IL-12 is also downregulated by the presence of *F. prausnitzii* that promotes production of IL-10 in homeostatic conditions,^102^ together with the expression of CD39, programmed death-ligand 1, and production of indoleamine 23-dioxygenase 1 (IDO-1) and IL-27. This dendritic cell phenotype can induce skewing of Th cells to Treg cells differentiation.^101^ IL-6 and TLR-4 expression levels on dendritic cells negatively correlated with that of *F. prausnitzii*, as well as IL-12p40 in same cells correlated with Bacteroides ratio.^103^

**Figure 3.**
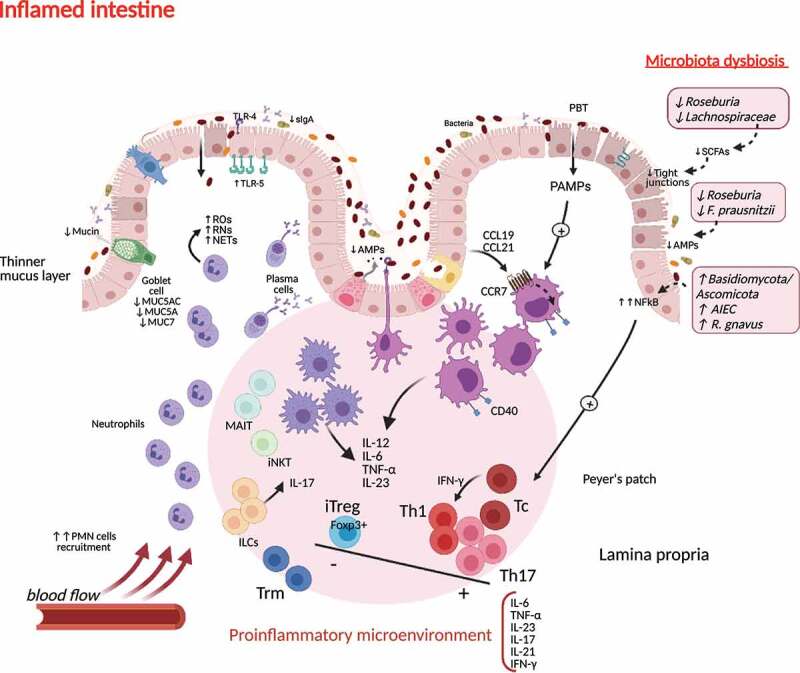
**Immunogenic microenvironment during microbiota dysbiosis in inflamed intestinal tissue in Crohn’s disease**. Crohn’s disease triggers induce a proinflammatory response orchestrated by tissue resident APCs activation, driving neutrophil recruitment, oxidative damage, the expansion of Th1 and Th17 populations and suppression of the regulatory T cell milieu. This environment favors the access of Tc and Trm cells to the damaged tissue. Innate lymphoid subpopulations cooperate in maintaining this immunogenic profile. Changes in gut microbiota composition and the reduction of SCFAs production contribute to the loss of tight junctions and the decrease of AMPs in the epithelial barrier, favoring an increased permeability (“leaky gut”). sIgA, soluble IgA; AMPs, antimicrobial peptides; ILC, innate lymphoid cells; MAIT, mucosal associated invariant T cell; iNKT, invariant Natural Killer T cell; DC, dendritic cell; iTreg, induced regulatory T cells; NFkB, nuclear factor kappa-B; Th, T helper cell; SCFAs, short-chain fatty acids, PMN, polymorphonuclear cells; TLRs, Toll-like receptors; Tc, T cytotoxic cell; NLRs, Nod-like receptors; IL, interleukin; ROs, reactive oxygen species; RNs, reactive nitrogen species; NETs, neutrophil extracellular traps; TNF-α, tumor necrosis factor-α; IFN-γ, interferon-gamma; CCL, chemokine ligand; CCR, chemokine receptor; PBT, pathological bacterial translocation; PAMPs; pathogen-associated molecular patterns; AIEC, adherent invasive *Escherichia coli.*

As previously stated, intestinal Paneth cells can sense bacteria by intracellular NOD-like receptors (NLRs), and they exert their control through production of antimicrobial peptides. Downregulation of antimicrobial peptides production may alter the composition of the microbiota and lead to an increase in susceptibility to inflammation^93^ (Figure 3). In fact, Paneth cells have a reduced production of α defensins in CD.^104^ Also, a reduction of mucosal-associated invariant T (MAIT) cells is observed in peripheral blood of CD, but an increase in their accumulation in intestinal inflamed areas. These MAIT cells can be activated by microbial-derived metabolites presented by dendritic cells in the context of MR-1 molecules and subsequently induce a proinflammatory tissue response.^105^

Moreover, the pathological variant of *E. coli* AIEC promotes Th17 differentiation in the intestine, and also remains functional inside macrophages, which elicit the release of high amount of TNF-α.^106^ This proinflammatory cytokine is also produced in a TLR-4 dependent way in response to *Ruminococcus gnavus*, a bacterial species related to CD among other inflammatory diseases.^16^ Also, the expression of PRRs TLR-5 in basolateral area of epithelium allows *Clostridium spp*. flagellin recognition, and this is one of the most representative antigens found in CD patients.^107^

As part of the innate immune response, innate lymphoid cell 3 (ILC3) subpopulation interacts with the intestinal microbiota and produces GM-CSF that helps maintain food tolerogenic responses and IL-22 that induces the production of antimicrobial peptides by epithelial cells. ILC3s express MHC-II that can control T CD4+ cell activation.^108^ In murine models, reduction of MHC-II in ILC3 induces inflammatory microbiota-dependent responses directed by T helper cells.^108,109^

Considering the adaptive intestinal T cell, Th CD4+ are highly abundant in the intestine and their response is mediated by crosstalk with local microbiota.^93^ Gut microbiota directs the balance of Th17 and Treg cells, and the altered response of T CD4+ to microbiota-derived antigens may give rise to a proinflammatory response in the intestinal microenvironment. Induction of Th1 and Th17 can be triggered by segmented filamentous bacteria in mice.^110^ These adherent bacteria can induce the production of IL-8/CCL20 chemokines and feedback the inflammatory response.^111^ Moreover, a biased microbiota-reactive Th17 response has been observed in IBD patients with a higher production of IL-17A.^112^ Blocking of IL-17A in CD patients has been showed to rise disease symptoms.^113^

In the case of fungal microbiota, the Basidiomycota/Ascomycota ratio has been shown increased in the intestinal mucosa of CD patients with active disease.^114^ Furthermore, *Malasezia* species have been probed to induce the production of inflammatory cytokines by dendritic cells in patients carrying the *CARD9S12N* gene allele SNP, worsening colitis in models for CD.^115^ Also, antibodies recognizing *Saccharomyces cerevisiae* mannan (ASCA) have been detected in approximately 50% of CD patients, while these antibodies are measured in around 8%–20% of the healthy subjects.^116^

## 4. Impact of the microbiota on fibrosis in CD patients

While at least 10% of CD patients have a clinically apparent fibrostenosing phenotype at diagnosis, the majority of patients initially exhibits a “pure” inflammatory phenotype without complications (strictures or fistulae).^117,118^ In population-based studies, the rate of patients progressing to fibrostenosis is approximately 20% at 20 years,^119^ a number reaching more than 30% within 10 years of diagnosis in tertiary referral centers.^117,118^ The location of strictures seems to be determined by the segmental location of inflammation, the most common site being the terminal ileum or the ileocecal region. Strictures, however, can occur in any segment of the intestine, including the upper gastrointestinal tract.^120^

Concepts on the pathogenesis of intestinal fibrosis in IBD and especially in CD patients have significantly changed over the last decade.^121^ For many years, intestinal strictures were seen as a consequence of long-standing inflammation.^122^ IBD-associated intestinal wall fibrosis was seen as an irreversible process frequently followed by stricture formations and subsequently intestinal obstruction requiring surgical intervention.^121^ However, despite the improved control of inflammation with new drugs such as biologics, the progression from inflammation to fibrosis in many CD patients has remained largely unaffected^123^ and the rate of surgery due to strictures remains high.^124^

The impact of the microbiota for tissue fibrosis was recognized in recent years and has been addressed by different experimental and clinical approaches. There seems to be both an impact of live bacteria as well as PAMPs. These microbial patterns, as well as “damage-associated molecular patterns” (DAMPs), bind or ligate to PRRs,^122^ thereby triggering tissue fibrosis. Interestingly, single-nucleotide polymorphisms affecting the function of PRRs and subsequently affecting bacterial sensing, recognition, or processing, such as the previously mentioned variants of NOD2, are associated with fibrostenotic CD.^55^

Mesenchymal cells such as collagen-forming fibroblasts express multiple PRRs such as TLRs and NLRs.^125^ Activation of those PRRs by bacterial wall products, bacterial DNA,, or proteins may drive mesenchymal cells into differentiation into a profibrotic phenotype. In addition, IECs may undergo epithelial-to-mesenchymal transition and contribute to intestinal fibrosis.^126^ The presence of EMT-associated molecules was demonstrated in fibrotic lesions of CD patients.^126^

Genetic variants encoding bacterial sensing PRRs as well as serum antibodies against microbial components have been associated with the risk of developing intestinal fibrosis.^121,125^ Animal models do not develop intestinal fibrosis in the absence of a microbiota.^125^ *In vitro* data suggest a specific action of flagellin in the profibrogenic response of intestinal mesenchymal cells.

**Figure 4.**
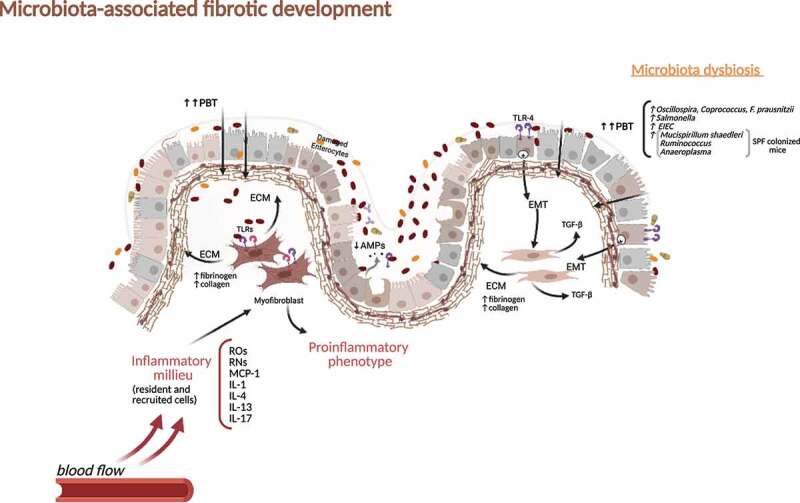
**Intestinal fibrosis development during microbiota dysbiosis in Crohn’s disease**. Mediators generated during sustained inflammation and continued PBT favored by gut barrier distortion induce myofibroblasts activation and extracellular matrix production with deposition of fibrinogen and collagen. Intestinal epithelial cells undergo EMT after activation of TLR-4 and contribute to the fibrotic context by inducing TGF-β. PBT, pathological bacterial translocation; TLRs, Toll-like receptors; ECM, extracellular matrix; EMT, epithelial to mesenchymal transition; ROs, reactive oxygen species; RNs, reactive nitrogen species; MCP-1, Monocyte Chemoattractant Protein-1; IL, interleukin; TGF-β, transforming growth factor-beta; SPF, Specific-pathogen-free; EIEC, Enteroinvasive *Escherichia coli.*

In a situation of a “leaky barrier,” translocation of PAMPs may occur through the gut epithelium, leading to the activation of subepithelial myofibroblasts via PRRs (e.g. TLR1 to 9 and Nod1 and −2)^125^ followed by NFκB activation and expansion of fibroblasts (Figure 4). TLR5 ligands are known to induce the fibroblast cell cycle entry and proliferation and IECs undergo EMT after activation of TLR4.^127^ However, fibrosis may also occur independent of the signaling of PRRs. In a heterotopic transplant mouse model of intestinal fibrosis, the deletion of MyD88, the adaptor protein of TLRs, necessary for signal transduction did not alter development of fibrosis. Collagen deposition and transforming growth factor-beta 1 (TGF-β1) expression was equal in MyD88+/+ and MyD88-/-, indicating that MyD88 was not essential for fibrogenesis.^128^

In addition to the discussion about a potential pathogenic role of bacterial proteins and bacterial wall compounds, there is evidence for a direct effect of certain microbes on fibrosis formation. When mice are infected and colonized with *Salmonella* (after pre-disposing streptomycin treatment), edema, mucosal ulcerations, and severe transmural inﬂammation is known to be a consequence of this colonization. However, besides inflammation, signiﬁcantly increased expression of transforming growth factor-B, connective tissue growth factor, and insulin-like growth factor-I has been detected.^129^ This leads to collagen type I deposition in the mucosa, submucosa, and muscularis mucosa and subsequent persistent intestinal wall fibrosis.^129^ Besides *Salmonella*, the CD-associated pathobiont entero-invasive *Escherichia coli* (EIEC) was reported to cause fibrosis in mice via a ﬂagellin-dependent mechanism via IL-33 induction and activation of the IL-33 receptor.^130^ Colitis-susceptible IL-10-deficient mice develop fibrosis when monoassociated with AIEC that harbors the yersiniabactin (Ybt) pathogenicity island.^131^ Inactivation of the Ybt siderophore production in AIEC nearly abrogated fibrosis.^131^ These findings may be specific for the genetic background of the host and the specific situation of IL-10 deficiency. On the other hand, it shows the impact of the microbiota on fibrosis development. This is further supported by the finding that the infection with chronic adherent-invasive *E. coli* in streptomycin-treated mouse strains (CD1, DBA/2, C3H, 129e and C57BL/6) induced profibrotic growth factors and fibrosis.^132^ Further evidence comes from another mouse model: tumor necrosis factor–like cytokine 1A (TL1A, TNFSF15) expression is increased in the inflamed gut mucosa and associated with fibrostenosing CD. Tl1a-overexpression in mice is followed by ileitis and fibrosis.^133^ When germ-free wild-type and Tl1a-transgenic mice were inoculated intragastrically with stool from specific pathogen–free (SPF) mice and a healthy human donor, the reconstitution with SPF, but not human microbiota, resulted in increased intestinal collagen deposition and fibroblast activation in the Tl1a-transgenic strain.^133^ Microbiota strains that were associated with increased fibrosis in this mouse model were groups of mucolytic bacteria such as *Mucispirillum schaedleri* and *Ruminococcus. Anaeroplasma* were also significantly associated with fibrosis in the cecum of SPF-colonized mice. *Oscillospira* and *Coprococcus* were negatively correlated with fibrosis in the cecum.^133^ Furthermore, similar to other models, *F. prausnitzii* and *Bacteroides* were negatively associated with fibrosis.^133^

To further elucidate the impact of the microbiota on the risk of intestinal stricture formation, we recently investigated the post-operative microbiota in CD patients that had already undergone resection of the ileo-cecal region or a segment or small intestine, with CD patients that had not needed an operation.^1^ Parabacteroides and Clostridiales were reduced in inactive postsurgical patients, and Enterobacteriaceae were significantly and reproducibly increased.^1^ All these findings point to a specific role of host–bacteria interaction for intestinal fibrosis. However, further research will be necessary to understand the mechanisms behind in more detail to be able to use this knowledge for therapeutic interventions.

## 5. Microbiota modulation targeting dysbiosis and potential beneficial effects in CD

Once reviewed the impact of the genetic background and the mucosal immunity dynamic influence in microbiota and disease progression, exogenous factors must be also considered when drawing the global picture of gut microbiota biological interactions and their relevance in CD pathogenesis.

Diet has substantial influence on the microbiome, intestinal permeability, and development of intestinal inflammation.^134^ For instance, western diet induces a shift in the microbiota composition, enhancing susceptibility to AIEC infection and intestinal inflammation.^135^ Also, smoking has been detected as the single-most important lifestyle factor impacting on the gut microbiome in CD patients and represents a well-accepted risk factor for a more complicated disease course in CD.^136^ In addition, exercise has been reported to deliver health benefit to CD patients,^137^ and in terms of impact on the microbiome associates with taxa also represented mainly in the CD cluster of taxa that if reduced in abundance associates with worse outcome. In other words, it is tempting to speculate that many lifestyle factors such as diet, smoking and exercise mediate their impact on the course of CD at least partly via changes in gut microbiota. Vice versa a change in diet, stop smoking and boosting exercise may well shift the microbiome to eubiosis and thus, at least contribute to achieve stable remission in CD.

The intestinal microbiota can be also modulated in order to improve the inflammatory response observed in CD, as well as considered a potential marker for disease state and progression. The knowledge of specific microbial drivers of pathogenic immune responses is crucial for achieving an effective microbial therapy.^138^ Rebalancing the intestinal microbiota through the enrichment of a specific anti-inflammatory bacterium, such as *F. prausnitzii*, is a potential strategy for CD treatment.^139^ An increase in Bifidobacterium, Collinsella, Lachnospira, Lachnospiraceae, Roseburia, and Eggerthella taxa has been linked to a strong response to anti-TNF-α treatment in CD.^1^ From a molecular point of view, the delivery of *L. lactis* containing a plasmid encoding for the anti-inflammatory molecule MAM has been shown effective in prevention of dinitrobenzene sulfonic acid-induced colitis in mice.^101^

Furthermore, the use of probiotics has been considered in CD. M2 macrophages, cells with immune anti-inflammatory properties, are induced by probiotic cocktail VSL#3 (four strains of *Lactobacillus*, three strains of *Bifidobacterium* and one of *Streptococcus*).^140^ This probiotic treatment helped to maintained remission in patients with CD.^141^ Dendritic cells with tolerogenic activities (CD103+) can induce inhibition of Th1/Th17 immune responses by promoting iTreg differentiation and by producing different intermediaries such as TGF-β or retinoic acid.^142^ Although IL-17 production is elevated in CD patients, treatment with antibodies anti-IL17 has been shown in the past to exacerbate the disease.^143^ However, microbiota products have been shown to ameliorate Th17 pathway, as polysaccharides from *B. fragilis* and protein fractions from *F. prausnitzii* can induce IL-10-producing Treg in mice.^97^

At present, fecal microbiota transplantation (FMT) in CD treatment is under study, with different clinical trials recently finished or in progress. Xiang *et al*. have reported clinical symptoms improvement in 43.7% of 174 CD patients treated and clinical remission in 20.1%.^144^ Also, Sokol *et al*. led a randomized controlled study of FMT in CD patients. They did not find differences in clinical remission for FMT-treated patients, but they observed that the incidence of flare in the FMT group was lower than in the control group of CD patients.^145^ Later, Li *et al*. noticed that adult population with the second dose of FMT at four months of receiving first, achieved long-term good clinical benefits.^146^ Moreover, a protocol for a double-blind, randomized, placebo-controlled pilot study in CD pediatric population is under development (Trial registration number NCT03378167).^147^

While conclusive clinical benefits yet require further study, expanding data on FMT anti-inflammatory effects are also emerging. The ongoing IMPACT-Crohn study^145^ shows a tendency toward decreased circulating white blood cells in fecal versus sham transplanted patients. In addition, *Burrello et al*. have reported the modulation of T cell phenotypes and the significant reduction of pro-inflammatory cytokines in colonic tissue after FMT transplant in a murine model of DSS-induced colitis.^148^

## 6. Future perspective

The modulation of intestinal microbiota and its communication with host’s regional and systemic immune behavior constitute a major research venue and a translational opportunity for improving CD outcomes. Considering that CD progresses mainly due to a sustained immune dysfunction and the central role of microbiota dysbiosis in negatively shaping immune homeostasis profile, the accurate accomplishment of microbiota eubiosis, bearing in mind the genetic, epigenetic, and environmental factors, is of outmost relevance for disease management.

In order to come to planned interventions, it becomes necessary to develop a much deeper mechanistical understanding of the ways microbiota and their derivatives contribute to mucosal immunity and how dysbiosis modifies their crosstalk, eventually comprising a CD pathogenesis hallmark. The identification of relevant specific bacterial metabolic pathways that may provide targeted interventions, as well as new insight on epithelial and immune cells biology, interaction and response to such highly dynamic microbiome, should be considered in all further research efforts.

The availability and use of new sophisticated technologies such as *in vitro* systems with complex organoids, analysis of gene expression, and epigenetic modifications regulating colonic tissue response or CRISPR-based new mutations screenings, among others, will help advance in our knowledge on immune mechanistic interactions with dysbiotic microbiota, their role in disease pathophysiology and how to intervene on them to personalize CD management.
